# Acute Kidney Injury in COVID-19 Patients Admitted at a Tertiary Care Centre: A Descriptive Cross-sectional Study

**DOI:** 10.31729/jnma.7931

**Published:** 2023-01-31

**Authors:** Diwakar Manandhar, Dhiraj Narayan Manandhar, Pramod Kumar Chhetri, Nishant Acharya, Rubash Nath Yogi, Anup Raj Upreti, Radhay Shyam Yadav, Nischal Shrestha, Sammi Joshi

**Affiliations:** 1 Department of Nephrology, Nepal Medical College and Teaching Hospital, Jorpati, Kathmandu, Nepal; 2 Clinical Pharmacist, Nepal Medical College and Teaching Hospital, Jorpati, Kathmandu, Nepal; 3 Department of Internal Medicine, Nepal Medical College and Teaching Hospital, Jorpati, Kathmandu, Nepal; 4 Kharanitar Primary Health Centre, Nuwakot, Nepal; 5 Nepal MedicafCollege and Teaching Hospital, Jorpati, Kathmandu, Nepal

**Keywords:** *acute kidney injury*, *COVID-19*, *Nepal*

## Abstract

**Introduction:**

Coronavirus disease can affect the renal system in various forms ranging from mild proteinuria to acute kidney injury, some even needing renal replacement therapy. This study aimed to find out the prevalence of acute kidney injury in patients admitted with COVID-19 at a tertiary care centre.

**Methods:**

This descriptive cross-sectional study was done in patients admitted in COVID-19 ward in our hospital from July 2021 to June 2022. Ethical approval was obtained from the Institutional Review Committee (Reference number: 066-077/078). The serum creatinine level was used for the diagnosis of acute kidney injury. Convenience sampling method was used. Point estimate and 95% Confidence Interval were calculated.

**Results:**

Out of 80 patients with COVID-19, the prevalence of acute kidney injury was 25 (31.25%) (21.09-41.41, 95% Confidence Interval).

**Conclusions:**

The prevalence of acute kidney injury in COVID-19 patients was similar to other studies done in similar settings.

## INTRODUCTION

A new strain of coronavirus was identified in December 2019 and was named severe acute respiratory syndrome coronavirus 2 (SARS-CoV-2).^1^ It was reported that SARS-CoV-2 interacts with the human angiotensin-converting enzyme II molecule, which is highly expressed in lung tissues and in human kidneys.^2,3^

Not only the lungs but SARS-CoV-2 also affects the human kidney resulting in hematuria, proteinuria and acute kidney injury (AKI), which is reversible injury to the kidneys.^4^ Patients with renal involvement have higher mortality than those without renal involvement.^5,6^

Worldwide there has been an extensive study regarding the disease. In Nepal, there has been a very limited study about the disease and there is a lot of information gaps regarding the disease.

The aim of the study was to find out the prevalence of acute kidney injury in COVID-19 patients admitted at a tertiary care centre.

## METHODS

This descriptive prospective study was carried out among patients with COVID-19 at Nepal Medical College Teaching Hospital, Kathmandu, Nepal over a period of one year from July 2021 to June 2022 after taking ethical clearance from the Institutional Review Committee (Reference number: 066-077/078). All the patients of ≥18 years of age with COVID-19 diagnosed by real-time polymerase chain reaction (RT-PCR) test were included in this study after taking informed consent. Patient with chronic kidney disease, renal transplantation or receiving maintenance hemodialysis and >14 days of radiological remission from COVID-19 were excluded from the study. Convenience sampling technique was used.

The sample size was calculated using the formula:


$$\begin{array}{ll} \text{n} & ={\text{Z}}^{2}\times \frac{\text{p}\times \text{q}}{{\text{e}}^{\text{2}}}\\ & =1.96^{2}\times \frac{0.28\times 0.72}{0.1^{2}}\\ & =78 \end{array}$$

Where,

n = minimum required sample size
Z = 1.96 at 95% Confidence Interval (CI)
p = prevalence from previous study taken as 28%^7^
q = 1-p
e = margin of error, 10%

The monimum sample size calculated was 78. However, the final sample size taken was 80.

AKI was diagnosed according to the Kidney Disease: Improving Global Outcomes (KDIGO) 2012, when any of these three criteria was present; an increase in serum creatinine by 0.3 mg/dl within 48 hours or a 50% increase in serum creatinine from baseline within 7 days or a urine volume of less than 0.5 mL/kg/hr for at least 6 hours.^8^ Clinically, we looked at serum creatinine for diagnosis, as proper collection and measurement of urine volume was not possible in all admitted patients.

Data collected from patient files included patient characteristics-age, gender, the date of onset of symptoms, comorbidities, complete blood count, C-reactive protein, Erythrocytes sedimentation rate, pro-calcitonin, urine R/M/E, serum urea, serum creatinine, serum sodium, serum potassium, serum calcium, serum phosphate, serum uric acid, ultrasonography (USG) abdomen pelvis, D-dimer, creatine phosphokinase, lactate dehydogenase, prothombin time/International normalized ratio (PT/ INR), Activated Partial Thromboplastin Time (APTT), and Ferritin.

Data were entered and analysed using IBM SPSS Statistics version 21. Point estimate and 95% Confidence Interval were calculated.

## RESULTS

Among 80 patients with COVID-19, acute kidney injury was seen in 25 (31.25%) (21.09-41.41, 95% CI). The mean age of the patients of COVID-19 with AKI was 52.08±17.50 years and the mean body mass index (BMI) was 24.52±3.69 kg/m^2^. Among 25 COVID patients with AKI, 8 (32.00%) was admitted in intensive care unit (ICU) and 17 (68.00%) in COVID ward. Regarding associated comorbidities, 5 (20.00%) were having diabetes and 6 (24.00%) hypertensions. Three (12.00%) gave history of previous kidney disease, 15 (60.00%) gave history of recent antibiotic use and 8 (32.00%) gave history of recent non-steroidal anti-inflammatory drugs (NSAIDs) use. Mean cycle threshold (CT) severity score was 12.76±4.7. Mean serum creatinine level on day 0 was 0.61±0.26, day 2 was 1.29±1.23 and day 7 was 1.98±3.09 mg/dl. Mean procalcitonin level was 0.85±1.80 ng/ml (Table 1).

**Table 1:** Baseline characteristics (n = 25).

Characteristics		n (%)
Admission	Ward	17 (68)
	ICU	8 (32)
History of Diabetes Mellitus		5 (20)
History of Hypertension		6 (24)
History of NSAIDs Use		8 (32)
History of Previous Kidney Disease		3 (12)
History of Antibiotics Use		15 (60)
CT Severity	Mild	6 (24)
	Moderate	10 (40)
	Severe	9 (36)

Among 25 COVID-19 patients with AKI, majority of the patients 14 (56.00%) had stage I, 7 (28.00%) had stage II and 4 (16.00%) had stage III AKI.

Five (20.00%) patients who had AKI underwent dialysis but could not survive giving mortality rate 6.25% (Table 2).

**Table 2:** Renal outcome of patients (n= 25).

Outcome	n (%)
Improved	20 (80)
Dialysis	5 (20)

## DISCUSSION

Among 80 patients the prevalence of AKI in COVID patients in our study was 31.25% similar to 36.84% reported by a study done in Nepal in 2021^9^ but higher than 10.60% reported in another study.^10^ This increase in rate is most probably due to availability of more sample for a proper study and more information regarding disease process. The cause of kidney involvement in COVID-19 is likely to be multifactorial, with cardiovascular comorbidity and predisposing factors (such as sepsis, hypovolaemia, and nephrotoxins) as important contributors.^11^

In this study, 24% patients were hypertensive, 20% was diabetic, 32% had recently used NSAIDs medications, 12% gave history of previous kidney disease which was similar to a study done in India.^12^ Their study showed a significant increase in the risk of severe COVID-19 (risk ratio of 2.11) compared with patients without comorbidities. But this study failed to show a positive correlation with severity, probably due to a small sample size.

In this study, 32% of cases with AKI was admitted in ICU due to severity of disease. It was similar to a study conducted in Florida in 2021.^13^ ICU mortality due to COVID-19 around the world and in the Unites States, in particular, have ranged from 20-62%.^13^ In this study 20% of AKI cases underwent dialysis who were admitted in the ICU and were under mechanical ventilation. but none could not survive. In mechanically ventilated patients, mortality has ranged from 50-97%.^13^ Based on these high mortality rates, there has been speculation that this disease process is different than typical ARDS, suggesting that standard ARDS mechanical ventilation strategies may not be as effective in reducing lung injury.

Unlike previous corona virus disease such as severe acute respiratory syndrome (SARS) CoV and Middle East respiratory syndrome (MERS) CoV, COVID-19 is a new strain of beta corona virus which created a worldwide pandemic affecting over 538 million people worldwide and 6.32 million deaths till date.^14^ Initially it was thought that COVID-19 primarily infects the respiratory system but as time progressed different study concluded that it has the capability to affect many systems both directly and indirectly leading to more severe manifestations and renal involvement is one of the common manifestations.

Kidney involvement is commonly seen in COVID-19 patients with clinical findings like mild proteinuria in hospitalized patients, while AKI develops at an advance stage in critically ill patients and is a marker of multiple organ failure and disease severity.^4^ Patients with AKI are more likely to have ICU admissions, have mechanical ventilation, and require vasopressors administration.^15^ In this study, 32% of cases with AKI was admitted in ICU due to severity of disease. Twenty percent of AKI cases underwent dialysis but could not survive.

Regarding pathophysiology of AKI, Ronco mentioned that COVID-19 pneumonia leads to right ventricular failure leading to kidney congestion and subsequent AKI. Similarly, left ventricular dysfunction might lead to low cardiac output, arterial underfilling, and kidney hypoperfusion.^11^ So, even if COVID is not directly linked to kidney disease, it can affect kidney physiologically in long term. Additionally, SARS-CoV-2 can directly infect the renal tubular epithelium and podocytes through an angiotensin-converting enzyme 2 (ACE2)-dependent pathway and cause mitochondrial dysfunction, acute tubular necrosis, the formation of protein reabsorption vacuoles, collapsing glomerulopathy, and protein leakage in Bowman's capsule. Another potential mechanism of AKI involves SARS-CoV-2-related immune response dysregulation, as indicated by observed lymphopenia and cytokine release syndrome (cytokine storm). Other contributors to AKI might include rhabdomyolysis, macrophage activation syndrome, and the development of micro emboli and microthrombi in the context of hypercoagulability and endotheliitis.^11^

The limitations of this study could be the sample size and sampling bias. Also, as this is a descriptive prospective study, we could not find association between variables and disease process. Further, this single-center study limits the generalizability of the findings to the whole Nepalese population.

## CONCLUSIONS

The prevalence of acute kidney injury among patients with COVID-19 was found to be similar to other studies done in similar settings. Recently the prevalence of COVID-19 cases is decreasing so indirectly number of patients with AKI will also decrease. Regardless of incidence, early recognition of kidney involvement in COVID-19 and early intervention is necessary to prevent subsequent AKI or its progression to reduce morbidity and mortality.
